# The Link Between Creativity, Cognition, and Creative Drives and Underlying Neural Mechanisms

**DOI:** 10.3389/fncir.2019.00018

**Published:** 2019-03-22

**Authors:** Radwa Khalil, Ben Godde, Ahmed A. Karim

**Affiliations:** ^1^Department of Psychology and Methods, Jacobs University Bremen, Bremen, Germany; ^2^Department of Psychiatry and Psychotherapy, University Clinic Tübingen, Tübingen, Germany; ^3^Department of Health Psychology and Neurorehabilitation, SRH Mobile University, Riedlingen, Germany

**Keywords:** creativity, cognitive flexibility, persistence, artistic shifts, emotion, reward, brain illness, neuromodulators

## Abstract

Having a creative mind is one of the gateways for achieving fabulous success and remarkable progress in professional, personal and social life. Therefore, a better understanding of the neural correlates and the underlying neural mechanisms related to creative ideation is crucial and valuable. However, the current literature on neural systems and circuits underlying creative cognition, and on how creative drives such as motivation, mood states, and reward could shape our creative mind through the associated neuromodulatory systems [i.e., the dopaminergic (DA), the noradrenergic (NE) and the serotonergic (5-HT) system] seems to be insufficient to explain the creative ideation and production process. One reason might be that the mentioned systems and processes are usually investigated in isolation and independent of each other. Through this review, we aim at advancing the current state of knowledge by providing an integrative view on the interactions between neural systems underlying the creative cognition and the creative drive and associated neuromodulatory systems (see Figure 1).

## Introduction

Creativity and innovative thinking have been a vast construct of questioning to scholars, psychologists, therapists and, more lately, neuroscientists (Jung et al., 2010). Creativity appears in various diverse models, tones, and shades (Feist, 2010; Perlovsky and Levine, 2012). The creative contributions of extraordinary artists, designers, inventors, and scientists attract our greatest consideration as they express the foundations of their culture and provide breakthroughs influencing cultural development and progress. Therefore, creativity is a crucial operator of human progress. Nevertheless, not every person who is an artist, inventor or scientist is similarly creative, nor are all creative (innovative) individual artists, inventors or scientists. Some are innovative in business, in communication with other individuals, or just in living.

**Figure 1:**
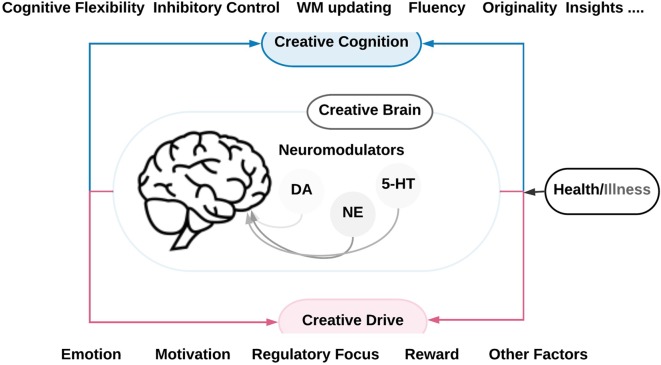
A schematic overview of the neurobiology of creativity as outlined in this review. It symbolizes the brain systems and neuromodulatory pathways underlying and modulating creative cognition and creative drive in health and disease. The creative cognition is based on various cognitive functions such as cognitive flexibility, inhibitory control, working memory (WM) updating, fluency, originality, and insights. The creative drive includes several factors that influence creativity such as emotion motivation, reward and other factors such as mood states, regulatory focus, and social interaction. The neuromodulatory pathways include the noradrenergic (NE), the dopaminergic (DA) and the serotonergic (5-HT) pathways.

Consequently, creativity is a multidimensional domain that could be executed in the arts, science, stage performance, the commercial enterprise and business innovation (Sawyer, 2006). Following Baas et al. (2015) who defined the roots of creative cognition in the arts and sciences, creativity is not just a cultural or social construct. Instead, it is an essential psychological and cognitive process as well (Csikszentmihalyi, 1999; Sawyer, 2006; Kaufman, 2009; Gaut, 2010; Perlovsky and Levine, 2012). Even so, many experimental investigations on creativity have reported various findings that often seem to be inconsistent and scattered. One of the principal reasons for that could be due to the wide variety of the experimental approaches in the domain of creativity research and the immense diversity in measuring and interpreting creative performance (Fink et al., 2007, 2014; Abraham, 2013; Zhu et al., 2013). In this review article we will discuss the relation between creative cognition, creative drives and their underlying neuromodulatory circuits (see Figures 1, 5 and Table 2). We will first elaborate on how different cognitive functions support creativity and on their neural basis as revealed by structural and functional brain imaging studies. Second, we will detail the link between mood and motivation as drives for creative performance and the role of dopamin (DA), noradrenaline (NE) and serotonin (5 HT) as key neuromodulatory systems. Next, we will discuss studies on pathological brain conditions which provide further evidence on the role of the neuromodulatory systems. Finally, based on this integrative view, we will list some open questions and provide suggestions for future research directions.

**Figure 2:**
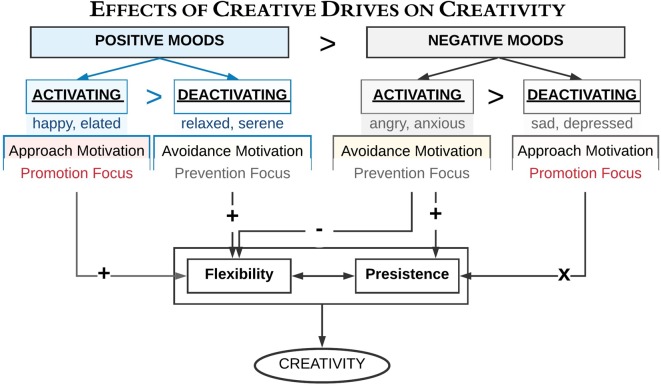
A schematic overview of the link between creativity and different mood states (after Baas et al., 2008, 2013; De Dreu et al., 2008). It illustrates how activating and deactivating mood states (i.e., valences, motivational state), and regulatory focus influence creativity. A “ >” symbolizes a higher influence in the condition left as compared to the right of the symbol. Symbols ± symbolize positive and negative influences, while an “X” symbolizes no influence revealed.

**Figure 3:**
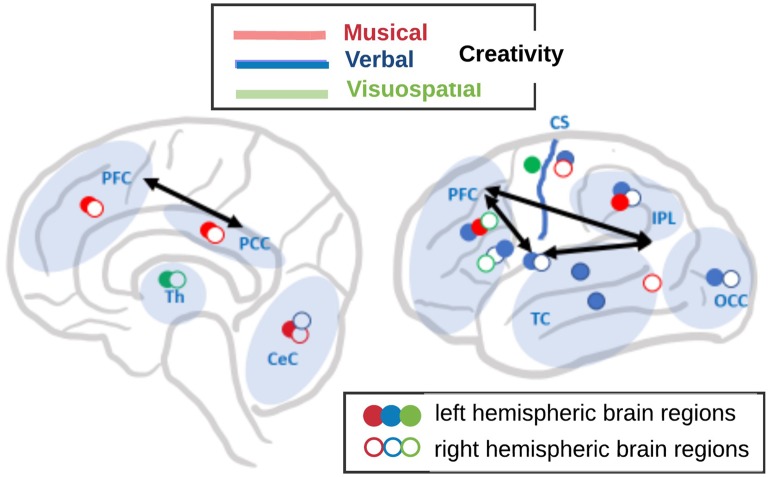
A schematic overview of the different networks in the brain involved in three dimensions of creativity (after Boccia et al., 2015): musical (red colored symbols), verbal (blue colored symbols), and visuospatial (green colored symbols). Filled symbols represent left hemispheric brain regions, open symbols represent right hemispheric regions. For simplicity, several separate foci within brain regions are represented by one single symbol. Brain regions are abbreviated as follows: PFC, prefrontal cortex; PCC, posterior cingulate cortex; IPL, intraparietal lobule; TC, temporal cortex; OCC, occipital cortex; Th, thalamus; CeC, cerebellar cortex; and CS, central sulcus. Black arrows symbolize the interaction between the executive control (EC) network and the default mode network (DMN) according to Beaty et al. (2017).

**Figure 4:**
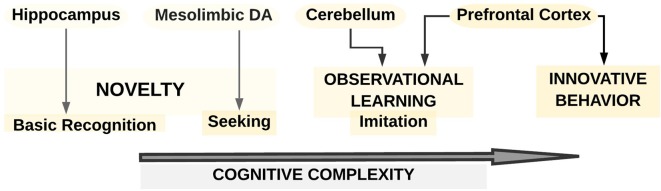
A schematic overview of the neurobiology of different facets of creativity as proposed from animal studies (after Kaufman et al., 2011). The creative animal model consists of three levels with increasing cognitive complexity: novelty, observational learning, and innovative behavior. The first level comprises of both the cognitive ability to recognize novelty, which is linked to hippocampal (HPC) function, and the seeking out of novelty, which is associated with the mesolimbic DA system. The second level refers to observational learning, which could range in complexity from imitation to the cultural transmission of creative behavior. Observational learning might critically depend on the cerebellum and the PFC. The third level is represented in the innovative behavior, which relates to specific recognition of a particular object characterized by novelty. This innovative behavior may be reliant upon PFC.

**Figure 5:**
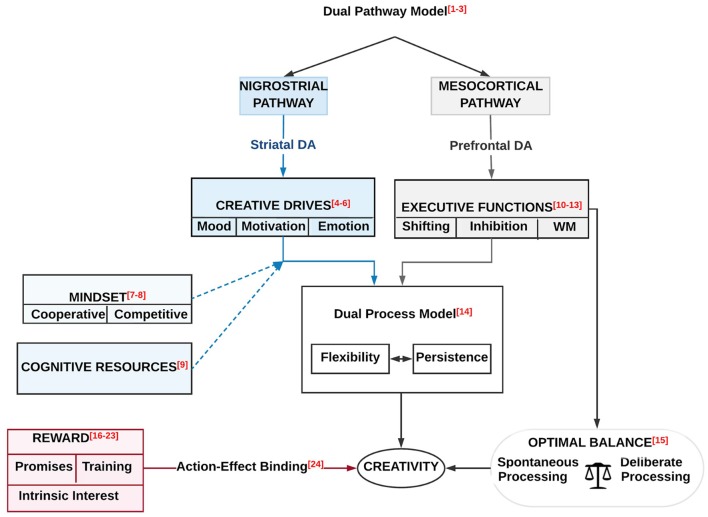
A schematic overview of the effects of the two DA pathways (the nigrostriatal and mesocortical DA) on the creative drives and the creative cognitions [i.e., executive functions (EFs)]. Both pathways influence creativity *via* the dual process model, which is composed of a resistance and cognitive flexibility. The prediction of creativity through EFs (i.e., shifting, inhibition and WM) requires an optimal balance between deliberate (controlled) processing and spontaneous processing. On the other hand, there is a link between reward (i.e., promises, training, and intrinsic interest) and creativity through the action effect binding. Moderating effects of mindset (cooperative and competitive) and cognitive resources on creative drives (i.e., mood, motivation, and emotion) is also illustrated. Numbers refer to references as indicated in Table 2.

**Table 1:** Potential candidate genes for creativity.

Authors	Potential Candidate Genes
Reuter et al. (2006)	DAT, COMT, DRD4, DRD2, TPH1
Zhang et al. (2014a)	DRD2
Zhang et al. (2014b)	COMT, COMT-DRD2
Zabelina et al. (2016)	DAT, COMT

**Table 2:** References related to corresponding numbers in Figure 5.

	References/Authors	Figure 5
[1–3]	(Nijstad et al., 2010; Boot et al., 2017; Lu et al., 2017)	Dual Pathway Model
[4–6]	(Baas et al., 2008, 2013; De Dreu et al., 2008)	CREATIVE DRIVES
[10–13]	(Benedek et al., 2014; Radel et al., 2015; Zhang et al., 2015; Fleming et al., 2016)	EXECUTIVE FUNCTIONS
[7–8]	(Bittner and Heidemeier, 2013; Bittner et al., 2016)	MINDSET
[9]	(Roskes et al., 2012)	COGNITIVE RESOURCES
[14]	(Cassotti et al., 2016)	Dual Process Model
[15]	(Mok, 2014)	OPTIMAL BALANCE
[16–23]	(Maltzman, 1960; Eisenberger and Selbst, 1994; Eisenberger and Cameron, 1998; Eisenberger et al., 1998, 1999; Eisenberger and Rhoades, 2001; Baer et al., 2003; Chen et al., 2012; Volf and Tarasova, 2013)	REWARD
[24]	(Muhle-Karbe and Krebs, 2012)	Action-Effect Binding

## Creative Cognition Is Rooted in Executive Functions (EFs)

The field of creative cognition deals with the understanding of the cognitive processes underlying creative performance. A pioneering study by Mednick (1962) linked creativity to associative thinking. This interpretation was not directed to any specific field of application such as art or science. Instead, it was attempted to define processes that underlie all creative thought. Rossmann and Fink (2010) extended Mednick’s theory by investigating the relationship between individual differences in processing associative information and various aspects of creativity.

Along with a variety of creative psychometric tasks, these authors provided a slightly modified variant of Gianotti et al.’s (2001) list of word pairs and asked the participants (university students) to rank the semantic associative distance between the words of a given pair. This list comprised pairs of indirectly related (e.g., cat—cheese) and unrelated word pairs (e.g., subject—marriage). In comparison to the less creative group, the more creative group reported smaller distances between unrelated word pairs, which can be interpreted as that they found creative associations between usually unrelated words.

Recently, Benedek et al. (2012) proposed a close connection between associative processes and divergent thinking (DT) as measured, for example, by the Alternate Uses Task (AUT, Guilford, 1967). Accordingly, the notion of creative cognition can be conceptualized within an evolutionary framework, namely Blind Variation and Selective Retention (BVSR; Jung et al., 2013). From a behavioral perspective, one could link the “blind variation” component to idea generation as measured by DT tasks. In contrast, the “selective retention” component could be represented by convergent thinking (CT), as represented by measures of remote associates (e.g., Remote Associates Test; RAT). Radel et al. (2015) revealed that inhibition influences certain kinds of creative processes selectively. Exposure to a Flanker or Simon task and thus exhausting inhibitory resources led to enhanced fluency and originality in a following AUT (i.e., DT) task. For a RAT (i.e., CT) task, no such effect was found (Radel et al., 2015). Therefore, a lack of resources for inhibition might lead to the facilitation of the frequency and the novelty (i.e., originality) of thoughts (i.e., ideas). Accordingly, one could claim that particularly idea generation processes profit from a depletion of the resources for inhibition.

Within a latent variable model approach, Benedek et al. (2014) explained the association between fluid intelligence and creative cognition through a general executive component. According to Benedek et al. (2014), creativity was predicted by working memory (WM) updating and inhibition, but not by mental set shifting. Further, WM updating and the personality factor openness represented a related factor of the shared variance between creativity and fluid intelligence (Benedek et al., 2014). Fleming et al. (2016) described associations between another personality trait, i.e., conscientiousness and mental set shifting, but not response inhibition nor WM updating. Level of conscientiousness influences whether people set and maintain long-range goals, deliberate over preferences (i.e., choices) or behave impulsively, and take obligations to others critically. It was associated with cognitive competencies which are related to rigid (i.e., inflexible) control over impulses (i.e., inhibition), and therefore might inhibit creativity. Mok (2014) highlighted the possibility for creative cognition to be originated from an optimal balance between spontaneous and controlled processes. It was hypothesized by Dietrich (2004) that the principal distinction between spontaneous and deliberate (i.e., controlled) modes of processing is the approach utilized to depict the unconscious novel information in WM. For example, the spontaneous process happens when the attentional system does not actively choose (decide or select) the content to become conscious, enabling unconscious thoughts that are relatively further random, unfiltered, and unusual to be represented in WM. On the other hand, deliberate insights are prompted by circuits in the prefrontal cortex (PFC) and therefore tend to be structured, rational (logical), and corresponding to internalized values and belief systems. A delicate balance between further spontaneous processing vs. more controlled processing may likely enhance creative cognition to the extent that default activity does not become suppressed due to the substantial need for controlled processing (Mok, 2012).

Cassotti et al. (2016) discussed how a dual-process model of creativity could expand our knowledge concerning the creative-cognitive associations. This dual-process model resembles the proposed model to account for reasoning and decision making (Evans et al., 1993). According to the dual pathway of creativity model (Nijstad et al., 2010), there are two qualitatively peculiar pathways to creative performance: the flexibility pathway and the persistence pathway. The flexibility pathway suggests stimulating creativity through a flexible switching between categories, approaches, and sets while the persistence pathway leads to creativity through hard work, systematic and effortful exploration of possibilities, and in-depth exploration of just a few categories (Nijstad et al., 2010). Lu et al. (2017) also revealed that cognitive flexibility could enhance two critical forms of creativity (DT and CT) by reducing the cognitive fixation, which, however, at the same time reduces the creative benefits of cognitive persistence. Combined, during the process of task switching, there is often an implicit tradeoff between flexibility and persistence (Nijstad et al., 2010). When task switching strengthens flexibility, it reduces persistence and* vice versa* (Lu et al., 2017). Also, supported and directed effort can further improve creative performance (e.g., Lucas and Nordgren, 2015).

Concerning inhibitory control, it is acknowledged that this executive function (EF) might be a core process involved in creative problem solving and idea generations (Cassotti et al., 2016). During generating creative thoughts, individuals of all ages (i.e., children, adolescents, and adults) tend to follow the path of least resistance. In the meantime, proposed solutions are constructed based on the most common and accessible information within a distinct specialty, which leads to a fixation effect. Given these points, the ability to think about the novel (original) ideas necessitates: (1) inhibiting spontaneous solutions, that cross to mind rapidly and unconsciously; and (2) exploring original (novel) ideas using a generative type of reasoning.

## The Link Between Mood States, Motivation, Reward, and Creativity

### How do Mood States Influence Creativity?

Creativity is a multifaceted construct, in which different moods influence distinct components of creative thoughts (Kaufmann, 2003). A remarkable study by Baas et al. (2008) explained how creativity is enhanced most by the positive mood states (see Figure 2); see also Bittner et al. (2016). Baas et al. (2008) pointed out that positive-activating moods with an approach motivation and promotion focus (e.g., happiness) activated creativity. On the contrary, negative-activating moods with avoidance motivation and a prevention focus (e.g., fear, anxiety, or even relaxation) correlated with lower creativity. Surprisingly, negative-deactivating moods together with approach motivation and a promotion focus (e.g., sadness) did not link with creativity.

Consequently, mood shifts are crucial in scaling creativity. Along the same line, De Dreu et al. (2008) argued that activating moods (e.g., anger, fear, happiness, elation) induce more creative fluency (i.e., number of ideas or insights) and originality (i.e., novelty) than deactivating moods such as sadness, depression, relaxation, and sereneness do (Figure 2; see also, Yang and Hung, 2015). According to De Dreu et al. (2008), activating moods could affect creative fluency and originality through enhancing cognitive flexibility when the tone is positive while enhancing persistence when the tone is negative (see also, To et al., 2015). Despite the previous findings, which related decreased creativity to an avoidance motivation and prevention focus when in a negative mood (Baas et al., 2008, 2013), an intriguing investigation by Roskes et al. (2012) explicated the contrary. For instance, they indicated that avoidance motivation could stimulate creativity through cognitive effort. However, this finding is incompatible with the dual process model of creativity (Nijstad et al., 2010), which suggests that both flexible and persistent processing styles could construct a creative output. In other words, avoidance motivation has often been related to decreased creativity since it elicits a relatively inflexible processing style (Baas et al., 2008, 2013). Adjusting these disagreements, Roskes et al. (2012) viewed that people with an avoidance-motivated behavior are not incapable of being creative; instead, they have to compensate for their inflexible processing style by a demanding and constrained processing. Therefore, it is a matter of compensation. Noteworthy, Roskes et al. (2012) reported that whether the individuals are avoidance motivated or approach motivated, their creativity could be enhanced under certain circumstances. These circumstances necessitate their creativity to be directed to a role for goal achievement, which motivates them to exert an additional effort of high-cost cognitive function.

Focusing on anxiety as another mood state that affects creativity, Byron and Khazanchi (2011) provided a meta-analytical study on the association between anxiety and creative performance (i.e., figural and verbal tasks). Anxiety was significantly and negatively related to figural and verbal creative performance. Using fMRI, Gawda and Szepietowska (2016) revealed that trait anxiety could slightly modulate neural activation during the creative verbal performance, notably, in the more complicated tasks. Additionally, there were significant variations in brain activation during the performance of more complex tasks between individuals with low anxiety and those with high anxiety. Also, Lin et al. (2014) reported how emotions shape different creative achievements (CAs). In their study, the positive emotional states reduced switch costs while enhancing the performance in DT and problem solving (i.e., performance in an open-ended DT test and a closed-ended insight problem-solving task).

Moreover, cognitive flexibility (as measured by a switching task) could have a mediating impact on the association between the positive emotion and the insight problem solving, but not between the positive emotion and DT. Bledow et al. (2013) revealed a strong influence of the dynamic interaction of positive and negative mood on creativity. Extraordinary creativity, for example, necessitates that a person should experience an episode of negative affect. This episode should be followed by a reduction in negative affect and an increment in positive affect. This process is termed “an affective shift.”

Concerning mindset, regulatory focus and creativity, Bittner and Heidemeier (2013) observed that mindsets have no direct control over creativity while prevention focus decreases subsequent creativity. They explicated that a cooperative mindset activates a promotion focus while a competitive mindset activates a prevention focus. Thus, prevention focus provides the indirect negative effect of competitive mindsets on creativity (Bittner and Heidemeier, 2013; Bittner et al., 2016).

### Does Reward Matter in the Case of Creativity?

A number of researchers highlighted the strong connection between reward and creativity (Eisenberger and Selbst, 1994; Eisenberger and Cameron, 1998; Eisenberger et al., 1998, 1999; Eisenberger and Rhoades, 2001; Baer et al., 2003; Chen et al., 2012; Muhle-Karbe and Krebs, 2012; Volf and Tarasova, 2013; see, Figure 5 and Table 2). In the following subsection, we will detail this relationship. Muhle-Karbe and Krebs (2012) highlighted the impact of reward on the action-effect binding, which underlies the ideomotor theory. They defined this theory as the formation of anticipatory representations about the perceptual outcomes of an action, i.e., action-effect (A-E) binding, thus, presenting the functional basis of voluntary action control.

A startling study proposed that reward training could improve generalized creativity (Maltzman, 1960; Eisenberger and Selbst, 1994; Figure 5 and Table 2). This enhancement requires the presence of a high degree of divergent thought and a reward. Eisenberger et al. (1998) argued that the assured reward improves creativity if there is an explicit positive relationship between creativity and reward (either currently or previously, i.e., it does not matter when). Besides, Eisenberger and Cameron (1998) focused on reward, intrinsic interest, and creativity. Herewith, the contribution of behavioral processes and cognitive-induced motivation represented possible determinants of the reward effects, which were crucial factors for enhancing creativity. Progressing in reward and creativity, Eisenberger et al. (1999) depicted the consequences of earlier experiences of a promised reward for creativity. They investigated how creativity (measured by a DT task) could be boosted by the distinction of a positive association between reward and creative novel performance. The demand for such novel performance in one task (whether associated with reward or not) established the promise of reward as a cue for creative performance. Herewith, the reward could either increase or reduce creativity depending on how it was supervised. As for the incremental effects of reward on creativity, Eisenberger and Rhoades (2001) questioned whether two-ways reward could enhance creativity. Based on their study, the reward required a contingent relation to creativity. This relation strengthened the extrinsic motivation. Hence, the expected reward for exceptional performance could boost creativity by enhancing the perceived self-determination and, consequently, the intrinsic interest. Later on, Chen et al. (2012) highlighted the interactive influences of the level and form of reward system design on group creativity, and how this interplay could assist in mastering the identified obstacles in the prior research.

Lastly, Volf and Tarasova (2013) argued about the impact of reward on the performance of creative verbal tasks. The promise of the monetary reward was favorable for creative thinking and original solutions. Interestingly, monetary reward-induced changes in brain oscillations, as measured with EEG, were characteristic of men but not women (i.e., a promise of a cash reward were correlated with EEG changes in men but not in women). For instance, in response to the monetary reward, men expressed an increase in both the θ2-rhythm asymmetry and the power of α rhythm. This finding reveals that women might refer to a tendency for a different effective strategy for processing verbal information to create a more original solution in the verbal task to receive a cash reward; thus, the promise of monetary reward is favorable for creative thinking and original solutions.

## Where Bright Ideas Are Produced in Our Brains

Concerning the neural correlates of creative cognition, a number of studies referred to the PFC as one of the chief brain areas for new idea generation and inhibition of prevalent solutions (Carlsson et al., 2000; Flaherty, 2005, 2011; Karim et al., 2010; Krippl and Karim, 2011; Mok, 2014; Cassotti et al., 2016). The prefrontal brain regions are known as components of a deliberate control brain network and inhibition controller, which is considered to be a central process for problem-solving and idea generation from adolescence to adulthood (Cassotti et al., 2016).

Dietrich and Kanso (2010) pointed out that creative thinking does not critically depend on a particular single mental process or specific brain region, and it is not mainly associated with right brains, defocused attention, low arousal, or alpha synchronization, as it also has often been hypothesized. Rather, Dietrich and Kanso (2010) proposed further subdividing creativity into different subtypes to make it traceable in the brain. In the same vein, a meta-analysis of 45 fMRI studies by Boccia et al. (2015), suggested that creativity depends on multi-component neural networks and that creative performance in three different cognitive domains (musical, verbal, and visuospatial; see Figure 3) rely on diverse brain regions and networks. Using general activation likelihood estimation (ALE) analyses, these authors revealed creativity-related clusters of activations in all four cortical lobes while the maximum activation of the individual ALE expressed distinct neural networks in each creative cognition domain as follows:

1. Musical creativity expressed activation in a bilateral network consisting of the bilateral medial frontal gyrus (MeFG) and posterior cingulate cortex (PCC), left middle frontal gyrus (MFG) and inferior parietal lobule (IPL), and the right postcentral gyrus (PoCG) and fusiform gyrus (FG), as well as bilaterally the cerebellum.
2. The network for verbal creativity was left-hemispheric dominated and comprised of several activation foci in the left MFG, inferior parietal lobule (IPL), SMG, middle occipital gyrus (MOG), and middle and superior temporal gyrus (MTG and STG), and the bilateral inferior frontal gyrus (IFG) and insula, and the right lingual gyrus (LG) and cerebellum.
3. Visuospatial creativity relied on a slightly right-hemispheric dominated network including activation foci in the right MFG and IFG, the left precentral gyrus (PrCG), and the bilateral thalamus.

Concerning underlying brain networks, Mok (2014) further pointed out that EEG data related to creative cognition often inferred widespread alpha synchronization (synchronized brain waves that occur at 8–12 cycles per second), particularly in posterior regions. Controlled processing may co-occur with spontaneous cognition—mediated by a subset of the default mode networks (DMNs; e.g., the angular gyrus (AnG) in the posterior parietal cortex (PPC), which has been frequently implicated in creative cognition; Mok, 2014). Subsequently, when the demand for controlled processing is substantially increased, the DMN may be suppressed. There is preliminary evidence suggesting an association between alpha synchronization and default-mode processing. Also, Andrews-Hanna et al. (2014) highlighted the interplay between the DMN, with the systems of executive control (EC) while regulating components of internal thought. Importantly, response inhibition (which underlies creative thought) demands dynamic interactions of large-scale brain systems (Beaty et al., 2016, 2017). Herewith the default mode and EC networks, which usually show an antagonistic relationship, tend to cooperate in enhancing creative cognition and thus artistic performance.

Regarding WM, Takeuchi et al. (2011) explored the association between brain activity during the N-back task as widely used WM paradigm (Jaeggi et al., 2010) and a psychometric measure of creativity (with a DT test). Through multiple regression analysis, Takeuchi et al. (2011) reported a significant and positive correlation between individual creativity and brain activity in the precuneus (a part of the superior parietal lobule in front of the cuneus in the occipital lobe) during a 2-back WM task but not during the non-WM 0-back task. This finding was coupled with task-induced deactivation (TID) in the precuneus (as part of the DMN, i.e., the brain network that is functional during the resting state), and correlated with higher DT. Using resting-state functional connectivity (RSFC) measures, Takeuchi et al. (2012) further showed an association between the medial PFC (mPFC) and PCC as the key nodes of the DMN during DT.

Another study revealed that DT was positively correlated with the strength of the RSFC between the mPFC and the MTG (Wei et al., 2014). Further, cognitive stimulation through creativity training significantly increased the RSFC between the mPFC and the MTG. Besides, cognitive stimulation successfully enhanced cognitive performance in a novelty (originality) creativity task (Wei et al., 2014).

An exciting study linked psychometric measurements of creativity [both DT and CA to cortical thickness in various brain regions in healthy young adults (Jung et al., 2010)]. In detail, these authors suggested the following: (1) higher CA was positively correlated with volume of the lower left lateral orbitofrontal cortex (lOFC) and cortical thickness in the right AnG; and (2) a composite creativity index (CCI) was negatively correlated with cortical thickness in the LG while positively correlated with cortical thickness in the right PCC.

Concerning the relation between hemispheric brain lateralization and creative thinking (i.e., formulating and producing novel ideas), a meta-analytic evaluation by Mihov et al. (2010) implied relative dominance of the right hemisphere (RH) during creative thinking. However, moderator analyses revealed no difference in predominant RH activation for many creative tasks (verbal, figural, holistic, analytical, context-dependent and context-independent). Carlsson et al. (2000) also analyzed the connection between creativity and hemispheric asymmetry, by measuring regional cerebral blood flow (rCBF) during rest and different creative verbal tasks. Highly creative subjects expressed bilateral frontal activation in the Brick task, a task in which participants were required to name potential uses of an object, while low creative subjects had unilateral activation. Importantly, in a word fluency test and the Brick test, the highly creative group expressed either an increase or unchanged CBF activity in the frontal region, while the low creative group showed a decrease in CBF instead.

Only a few animal studies also provided valuable insights into the link between brain and creative cognition. For example, a framework developed by Kaufman et al. (2011) suggested a three-level model of creativity (novelty, observational learning, and innovative behavior; see Figure 4). First, regarding novelty, the cognitive ability to recognize was proposed to be linked to hippocampal (HPC) function while seeking out for novelty could be connected to the mesolimbic DA system. Second, observational learning, which could range in complexity from imitation to the cultural transmission of creative behavior, was supposed to rely significantly, besides frontal brain regions, on the cerebellum. Third, innovative behavior such as creating a tool or exhibiting a behavior with the specific recognition that it is novel and different was described as being especially reliant upon the PFC and the balance between left-and-right hemispheric functions.

## How the Neuromodulatory Systems Are Involved in Creative Performance

### The Dopaminergic (DA) System and Creativity

The DA system is involved in various aspects of cognitive functions related to reward, addiction, attention, compulsions, and others. Recent studies imply that the DA system may act to coordinate the integration of information through selective potentiation of circuits and pathways (Grace, 2010). Several lines of evidence support the crucial role of DA neurotransmission in human creative thought and behavior (Flaherty, 2005; Reuter et al., 2006; Kulisevsky et al., 2009; Chermahini and Hommel, 2010; de Manzano et al., 2010; Inzelberg, 2013; van Schouwenburg et al., 2013; Lhommée et al., 2014; Surmeier et al., 2014; Zhang et al., 2014a,b, 2015; Zabelina et al., 2016; Boot et al., 2017; Kleinmintz et al., 2018), nevertheless, these studies remain sparse.

For example, Flaherty (2005) reported that novelty seeking and creative drive are influenced by mesolimbic DA. Colzato et al. (2009) measured spontaneous eye-blink rates (EBR) as a marker of central DA functioning in a stop signal task. They found that EBR predicted the efficiency in inhibiting tendencies to undesired action in this task. As these findings were obtained from patient and drug studies, the authors constrained their conclusions on a positive effect of DA stimulants on response inhibition to cases of suboptimal inhibitory functioning (Colzato et al., 2009). Later, Chermahini and Hommel (2010) revealed that EBR predicted flexibility in both kinds of thinking (DT and CT) but in different ways. Notably, there was a positive correlation between CT and intelligence, but a negative correlation with EBR, proposing a correlation between CT impairment and higher levels of DA.

Furthermore, Zhang et al. (2015) investigated the relation between EBR and many EFs (i.e., mental set shifting, response inhibition, and WM updating). Their study revealed a correlation between increasing EBR (which refers to increasing DA) with a better mental set shifting and response inhibition, but poorer WM updating. The increment in EBR levels was associated with an increase in the accuracy in both mental set shifting and response inhibition related tasks; however, a reduction in the cost of mental set shifting and response inhibition was associated with a decrease in the accuracy in WM updating tasks. These findings indicate a diverse role of the central DA system in mental set shifting and response inhibition as compared to updating (Figure 5; see also Zhang et al., 2017).

Recently, Boot et al. (2017) provided an integrative review on creative cognition and DA modulation in frontostriatal networks (see, Figure 5 and Table 2). Integrating results from different experimental tasks (i.e., creative ideation, DT, or creative problem-solving) and various study approaches (such as looking at polymorphisms in DA receptor genes, measuring indirect markers of DA activity, manipulating the DA system, or investigating clinical populations with dysregulated DA activity) proposed the followings: (i) creative cognition benefits from both flexible and persistent processing; (ii) an association between striatal DA, the integrity of the nigrostriatal-DA pathways, and flexible processing; and (iii) an association between prefrontal DA, the integrity of the mesocortical-DA pathway and persistent processing (Figure 5 and Table 2). Altogether, while the literature indicates a functional differentiation between the striatal and prefrontal DA, it seems that the functional level of DA has to be moderate for both striatal DA and prefrontal DA to benefit creative cognition by facilitating flexible processing and enable persistence-driven creativity, respectively (Boot et al., 2017).

## Regional Gray Matter Volume (rGMV) of The Dopaminergic (DA) System and Creativity

Despite the existence of a consistent number of functional imaging studies on creativity, the relationship between individual creativity and volumetric morphological changes in the regional gray matter (rGMV) within the DA system has not been explored adequately until recently. Salgado-Pineda et al. (2003) reported increased rGMV in parts of the mesencephalic DA system (thalamic, inferior-parietal, and frontal cortical regions) following the treatment with of levodopa (i.e., DA replacement therapy). Moreover, different studies on patients with Tourette’s Syndrome (which is another disease associated with an excessive function of the mesencephalic DA system) described related increases of rGMV in these regions (Shapiro et al., 1989; Singer et al., 2002; Albin et al., 2003). These investigations imply that the morphology of the mesencephalic DA system and associated DA function are correlated with creativity. This assumption is further supported by Takeuchi et al. (2010) who revealed a positive correlation between individual creativity (as measured by a DT task) and rGMV in particular parts of the mesencephalic DA system [i.e., the right dorsolateral PFC (rDLPFC), bilateral striata and anatomical clusters in the Substantia Nigra (STN), the ventral tegmental area (VTA) and periaqueductal gray (PAG)]. These findings resonate the core link between individual creativity and rGMV of the mesencephalic DA system. Accordingly, there is an agreement with the opinion that associates DA physiological mechanisms and individual creativity.

## Artistic Style Shifts, Dopamine (DA), and Creativity

An exciting study by Kulisevsky et al. (2009) described the relationship between mental shifts and the artistic style in Parkinson’s disease (PD) focusing on the link between creativity and DA. They provided a case study with a PD patient, which reported changes in the creative artistic performance. These changes appeared to be correlated with the DA imbalance in the limbic system. When this patient was supplied with DA agonists, then, hidden creativity had been awaked. This awake led to progressive improvement in painting productivity. Then, the rebirth of artistic creativity in PD relied on sustaining DA level (see also Inzelberg, 2013). However, it is yet unclear whether the enhancement of the creative drive was due to the physiological regulation of DA because the underlying mechanisms remain speculative (Inzelberg, 2013). It is well known that neurodegenerative diseases are characterized by reduced flexibility, conceptualization, and visuospatial abilities (Asaadi et al., 2016). Although these features are essential elements for creativity, case studies revealed the evolution of creativity during PD.

Along with the same line, Lhommée et al. (2014) explained the possibility of inducing creativity through DA treatments in PD; however, this effect feasibility slowly disappeared after withdrawal of DA agonists, and only one of eleven patients remained creative after the surgery. Also, the reduction of DA agonist was significantly correlated to the decrease in creativity in the whole study population. Consequently, there is a strong link between creativity in PD and DA agonist therapy.

## Genetic Research Reveals a Strong Association Between DA Activity and Creativity

One critical step towards a better understanding of creativity is to unveil its underlying genetic architectures. Many studies reported the first candidate genes for creativity (Reuter et al., 2006; Runco et al., 2011; Zhang et al., 2014a, b; Zabelina et al., 2016; Grigorenko, 2017; see Table 1).

On describing the genetic basis of creativity and ideational fluency, Runco et al. (2011) referred to Reuter et al. (2006) who defined what they called the first candidate gene for creativity. Runco et al. (2011) replicated and extended the investigation of Reuter et al. (2006) for further accurate analysis of five candidate genes, which are: DA transporter (DAT), catechol-O-methyl-transferase (COMT), Dopamine Receptor D4 (DRD4), D2 Dopamine Receptor (DRD2), and Tryptophan Hydroxylase 1 (TPH1). In the study by Runco et al. (2011), participants received a battery of tests related to creativity. Multivariate analyses of variance indicated a significant association between the ideational fluency scores and several genes (DAT, COMT, DRD4, and TPH1). Therefore, in contrast to initial studies, the offered conclusion by Runco et al. (2011) suggested a clear genetic basis for ideational fluency. However, fluency, alone, is not sufficient to predict and guarantee creative performance.

Mayseless et al. (2013) reported an association between DT and DRD4 (7R polymorphism in the DRD4 gene). DT abilities were associated with DA activity while impaired DT has been reported in populations with DA dysfunctions. The authors concluded that individuals carrying the DRD4–7R allele scored significantly lower in DT (particularly on the flexibility dimension) compared to non-carriers of this allele.

Zabelina et al. (2016) observed that performance in two tests of creativity (i.e., the Torrance test and the real-world CA index) could be predicted by specific genetic polymorphisms that are related to the frontal (COMT gene) and striatal (DAT gene) DA pathways. High performance at the Torrance test was related to DA polymorphisms associated with higher cognitive flexibility and low to medium top-down control (9/9 or 9/10 DAT and Met/Val or Val/Val COMT genotypes, respectively), or, particularly for the originality component of the DT, with weak cognitive flexibility and strong top-down control (10/10 DAT and Met/Met COMT genotypes, respectively). Weak cognitive flexibility (10/10 DAT genotype) and weak cognitive control (Val/Val COMT genotype) were associated with high real-world CA.

An additional exploratory study on DA gene DRD2 and the creative potential (DT test) was provided by Zhang et al. (2014a). This study systematically explored the associations between DRD2 genetic polymorphisms and DT in 543 unrelated healthy Chinese undergraduate students. There were significant associations between specific single-nucleotide polymorphisms (SNPs), fluency (verbal and figural), verbal originality and figural flexibility. Extending on these findings, Zhang et al. (2014b) thoroughly examined the relationship between COMT, creative potential and the interaction between COMT and DRD2. Their study provided a shred of evidence for the implication of COMT in creative potential, which suggests that DA-related genes may act in coordination to contribute to creativity.

Based on these findings, one can conclude that human creativity principally relies on the interplay among frontal and striatal DA pathways. The dynamical interaction between these two pathways might assist to explain the inconsistencies due to the independent evaluation in measuring genes and creativity during the past decade.

## Other Neuromodulatory Systems and Creativity

According to Flaherty (2011), the induction of creativity could rely on the goal-driven approach motivation from the midbrain DA system; however, fear-driven avoidance motivation could have an insignificant influence on creativity. Therefore, one could argue about the role of other neuromodulators in addition to DA regarding their influences on motivational behavior and creativity.

Researchers observed that when 5-HT and NE lower motivation and flexibility, they can inhibit creativity. For example, antidepressants (ADs) that inhibit fear-driven motivation (i.e., selective serotonin reuptake inhibitors) could inhibit goal-oriented motivation as well. On the other hand, ADs that boost goal-directed motivation (i.e., bupropion) may remediate this effect. As for benzodiazepines and alcohol, they might have a counterproductive effect. Although DA agonists might stimulate creativity, their actions may inappropriately disinhibit this creative behavior through suppressing its motivational drive. Moreover, it was suggested that the presence of NE induces fluctuations in levels of other catecholamines, such as DA, which has been extensively discussed in the schizophrenia literature.

## Noradrenaline (NE) System, and Creativity

The link between the noradrenergic (NE) system, arousal and the creative process has been examined either through the direct pharmacological manipulation of the NE system, or by investigating the influences of endogenous changes in the NE system (i.e., sleep and waking states) on behavior and cognition (Folley et al., 2003). Also, situational stressors correlate with particular physiological responses, including an increase in the activity of the NE system (Ward et al., 1983; Kvetňanský et al., 1997).

Experimental evidence proposed a central role of the NE system in modulating cognitive flexibility (Beversdorf et al., 1999, 2002; Folley et al., 2003; Heilman et al., 2003; Heilman, 2016; de Rooij et al., 2018). Beversdorf et al. (1999, 2002) investigated the influence of NE modulation on the performance in various problem-solving tasks during pharmacological treatments that either increased or decreased noradrenergic activity. The authors reported better performance in the anagram task (one of the problem-solving tasks that demand cognitive flexibility), following the uptake of propranolol (peripheral and central β-adrenergic antagonist) than after ephedrine (β-adrenergic agonist). Comparing the effects of central and peripheral NE antagonists, Beversdorf et al. (2002) further revealed that NE modulation of cognitive flexibility, in particular in problem-solving tasks, occurs by a central feedback mechanism. This is in agreement with an earlier reported influence of arousal on cognitive flexibility during creative tasks through the regulation of the central NE system (Martindale and Greenough, 1973). Martindale and Hasenfus (1978) provided physiological evidence about enhancing creative innovation through maintaining a low level of arousal (i.e., the significant development of alpha activity in the EEG in the highly creative group during the innovative stage). Also, the reported central modulatory effect of NE on cognitive flexibility may relate to changes in the signal-to-noise ratio of neuronal activity within the cortex by suppressing the intrinsic excitatory synaptic potentials relative to the evoked potentials by external direct afferent input (Hasselmo et al., 1997; Usher et al., 1999).

In light of the findings described previously (Hasselmo et al., 1997; Beversdorf et al., 1999, 2002; Usher et al., 1999), one could evaluate the dependency of problem-solving on the regulation states of the NE system. The first state refers to situations up-regulating the NE system, which diminishes cognitive flexibility while the second state relates to situations down-regulating NE system, which enhances cognitive flexibility.

For example, NE upregulation by increased situational stress could weaken cognitive flexibility and thus creativity (Beversdorf et al., 1999, 2002) while people seem to be highly creative during relaxation as compared to when they are stressed (Faigel, 1991).

Recently, de Rooij et al. (2018) explored the function of the LC-NA system in creativity using pupillometry. LC is a brain area which contains noradrenergic (NE) neurons that project to the frontal lobe modulating the frontal lobe’s activity (Arnsten and Goldman-Rakic, 1984). Accordingly, elevation in LC activity is correlated with increasing levels of cortical NE. de Rooij et al. (2018) now examined whether tonic pupil dilation and phasic pupil dilation (as proxies for measuring tonic and phasic LC-NA activity, respectively) could predict performance on divergent and CT using both psychometric and real-world creativity tasks. During DT, the tonic pupil dilation predicted the generation of original ideas in both creativity tasks while phasic pupil dilation predicted the generation of useful ideas only in the real-world creativity task. Nevertheless, during CT, tonic and phasic pupil dilation did not predict creative task performance in both creativity tasks. Hence, tonic and phasic LC-NA activity differentially predicted the generation of original and useful ideas during creative tasks that require DT.

## Serotonergic (5-HT) System and Creativity

The neurotransmitter serotonin [5-hydroxytryptamine (5-HT); Walther et al., 2003] is causally involved in multiple central nervous facets of mood control and in regulating sleep, anxiety, alcoholism, drug abuse, food intake, and sexual behavior (Veenstra-VanderWeele et al., 2000). Volf et al. (2009) provided one of the earliest reports on a significant association between the polymorphism in the human serotonin transporter gene [i.e., serotonin-transporter-linked polymorphic region (5-HTTLPR)] and CAs (i.e., figural and verbal). Up to now, however, there has not been sufficient evidence to conclude on a direct connection between 5-HT and creativity, but there has been between 5-HT and reward. Kranz et al. (2010) presented an argument regarding 5-HT as an essential mediator of emotional, motivational and cognitive elements of reward representation. Consequently, one could claim that 5-HT is of a similar value to DA for reward processing; nevertheless, it is mostly ignored in the studies related to creativity.

## Brain Illness and Creativity

Accumulated evidence suggests a strong connection between developing the drive of creativity and a number of brain illnesses (i.e., depression, bipolar disorder, psychosis, PD, temporal lobe epilepsy (TLE), frontotemporal dementia (FTD), and autism spectrum disorders (ASDs); see Flaherty, 2011, see also Flaherty, 2005; Carson, 2011; Abraham et al., 2012; Mula et al., 2016), other studies questioned the relation between madness and genius (Kyaga, 2014).

Flaherty (2005) tested a wide range of subjects from normal to several pathological states and proposed a three-factor model to predict idea generation and creative drive. This model focused on the interactions between temporal lobes, frontal lobes, and the limbic system, in which the frontotemporal and DA control represents the source for idea generation and creative drive. The author summarized her findings as follows. First, the generation of the progressive idea (sometimes at the expense of its quality) is associated with alterations in the activity of the temporal lobe (i.e., hypergraphia). Second, deficits in the frontal lobe might diminish idea generation due to the rigid judgments about the value of the idea. These observations were most visible in verbal creativity, and approximately resemble the constrained communication of temporal lobe epilepsy (TLE), mania, and Wernicke’s aphasia, rather than the sparse speech and cognitive inflexibility of depression, Broca’s aphasia, and other frontal lobe lesions. Third, patients with FTD expressed an enhancement in non-linguistic creativity. Lastly, the mutual inhibitory cortico-cortical interactions mediated the proper balance between temporal and frontal activity (Flaherty, 2005).

Abraham et al. (2012) examined distinct facets of creative thinking in many neurological populations as compared to matched healthy control participants. They reported a dissociation between patient groups with frontal, temporoparietal, and basal ganglia (BG) lesions for diverse aspects of creativity. The temporoparietal and frontolateral groups expressed lower overall creative performance while the temporoparietal group demonstrated reduced fluency in the AUT and a creative imagery task. On the other hand, the frontolateral group was less proficient at producing original responses. In contrast, BG and frontopolar groups showed remarkable performance in the ability to overcome the constraints demand by salient semantic distractors during generating creative responses.

Consequently, the lesion area posed selective obstacles to the ability to generate novel (original) responses in distinctive contexts, but not on the ability to generate relevant responses (which was compromised in most patient groups). Thereby, Mula et al. (2016) discussed FTD and bipolar cyclothymic mood disorder as clinical conditions that are assisting to unravel the underlying neuroanatomy and neurochemistry of human creativity. They described the emergence of artistic talent in a subset of patients with dementia who developed incipient and impassioned abilities in visual arts. Earlier, Miller and Miller (2013) stated that in addition to the emergence of visual artistry in such patients, new onset creativity occasionally extends to obsessions with word punning and poetry. These recently compelling artistic and creative behaviors have been noticed initially in non-Alzheimer’s dementia, specifically, those with primary progressive aphasia (PPA), a particular form of FTD (Wu et al., 2015; Mula et al., 2016). Furthermore, de Souza et al. (2014) reported a series of clinical observations about patients with neurodegenerative diseases affecting PFC (i.e., FTD) and the facilitation of artistic production.

On the link between creativity and bipolarity, researchers aimed at dissecting principal components of mania showing that feeling creative is usually told by patients with bipolar disorders (Cassano et al., 2009; Mula et al., 2016). These patients often express themselves as very artistic and creative with bursts of inspiration or creativity and mentally very sharp, brilliant and talented. Remarkably, specialized studies that focus exclusively at creativity in patients with mood disturbances explicated that even when using quite a broad definition of creativity, no more than 8% of patients with bipolar or unipolar disorders could be considered creative (Akiskal et al., 1998; Mula et al., 2016).

On the association between creativity and psychopathology, Carson (2011) provided an advanced model of a shared vulnerability to intensify creative ideation. This model suggested an interaction between the biological determinants, presenting the risk for psychopathology, and the protective cognitive factors. The elements of shared vulnerability included the following: (1) cognitive disinhibition (it brings more stimuli into conscious awareness); (2) an attentional style (which is driven by novelty salience); and (3) a neural hyperconnectivity (which may increase associations between diverse stimuli). These vulnerabilities interact with superior meta-cognitive protective factors (i.e., high IQ, increased WM capacity, and enhanced cognitive flexibility) to maximize the range and the depth of stimuli. Hence, stimuli, which are acquirable in conscious mindfulness, could be manipulated and integrated to form novel (original) ideas.

## Open Questions and Future Directions

The PFC, which is considered to play a critical role in creativity, has been extensively involved in the cognitive control of emotion; however, the cortico-subcortical interactions that mediate this capability remain elusive, in particular when it is related to creativity. Previously, Wager et al. (2008) declared that prefrontal-subcortical pathways mediate effective emotion regulation. This regulation was associated with the activity of the right ventrolateral prefrontal area (vlPFC) as a response to diminished negative emotional experience during cognitive reappraisal of aversive (i.e., unpleasant) images. Following this initial finding, researchers implemented a unique pathway-mapping approach to map subcortical mediators of the association between vlPFC activity and reappraisal achievement (i.e., a decrease in the expressed emotion). Their data proposed two distinct pathways that collectively defined half of the revealed variance in self-stated emotion. The first pathway [which was through nucleus accumbens (NAc)] anticipated more reappraisal achievement while the second pathway (through ventral amygdala) anticipated reduced reappraisal achievement. Here, one could ask whether the interaction between emotion and creative cognition could be predicted through similar pathways.

Regarding providing an overarching experimental model for creative performances, one should consider the interactions between the factors described in this review (cognition, emotion, mood state, reward, and neuromodulators) and whether such interactions could mark creative signatures of individuals. In other words, getting more insight into the creative thinking and ideation necessitates the ability to identify: (1) the core cognitive, motivational, and emotional processes underlying creative thought; and (2) the brain circuitries and neuromodulators underlying the creative ideation.

Prospective research should further specify the neural mechanisms by which the neuromodulator systems influence the creative process. Particularly their modulatory effect on the creative cognition and the creative drive in pathological conditions such as depression, bipolar disorders, PD and schizophrenia remains elusive. DA requires additional exploration regarding the interplay between frontal and striatal DA pathways, the underlying genetic architecture and CAs in healthy and pathological conditions. On the other hand, research on creativity and the noradrenergic (NE) system is implicated in the stress-related modulation of cognitive flexibility in problem-solving, however there is a prominent demand to determine the range of cognitive tasks modulated by the NE system more precisely. Also, studies on the relation between the fluctuations in the level of NE, the level of arousal and its modulation signature on the creative process before and after treatment in pathological conditions such as depression, bipolar disorders, and schizophrenia remain dispersed and isolated. Concerning 5-HT, there is an ultimate need for elaborative research on the relationship between 5-HT and CAs since it is a fundamental mediator of emotional, motivational and cognitive elements of reward processing and representation.

In summary, advancing the research on creativity demands providing an integrative framework assembling the neural, cognitive, motivational, and emotional correlates of creativity. Furthermore, computational approaches such as neural network models could assist to provide a predictive perspective for this integrative framework for creativity (Perlovsky and Levine, 2012). Although these models are not likely to be achieved merely, computational approaches to particular emotional processing could be both plausible and useful to develop the integrative framework model. For instance, Levine and Perlovsky (2011) proposed a dual-system approach to integrating emotional and rational decision making while Perlovsky and Levine, 2012 suggested a model of DA influences on creative processes. Thus, extending these computational models would be beneficial as a predictive approach to our proposed integrative framework for creativity.

## Conclusion

In this review, we outlined how three factors crucially shape the creative mind: (1) creative cognition and the associated neural systems in human and animal models; (2) creative drives such as mood states, emotion, motivation and regulatory focus and how their interactions could shape the creative performance; and (3) the impacts of three central neuromodulator systems, i.e., DA, NE, and 5-HT, on the interplay between creative cognition and creative drives.

Specifically, we detailed how according to the dual pathway model (Nijstad et al., 2010; Boot et al., 2017; Lu et al., 2017) the nigrostriatal and mesocortical DA pathways, influence creative drives (Baas et al., 2008, 2013; De Dreu et al., 2008) and creative cognition, see Figure 5 and Table 2. As implicated by the dual process model, both pathways affect creativity *via* their influence on resistance and cognitive flexibility (Cassotti et al., 2016). The prediction of creativity through EFs (i.e., shifting, inhibition and WM; Benedek et al., 2014; Radel et al., 2015; Zhang et al., 2015; Fleming et al., 2016) demands an optimal balance between deliberate (controlled) processing and spontaneous processing (Mok, 2014). On the other hand, there is a link between reward (i.e., promises, training, and intrinsic interest; Maltzman, 1960; Eisenberger and Selbst, 1994; Eisenberger and Cameron, 1998; Eisenberger et al., 1998, 1999; Eisenberger and Rhoades, 2001; Baer et al., 2003; Chen et al., 2012; Volf and Tarasova, 2013) and creativity through the action effect binding (Muhle-Karbe and Krebs, 2012). Both mindset (cooperative and competitive; Bittner and Heidemeier, 2013; Bittner et al., 2016) and cognitive resources (Roskes et al., 2012) have moderating effects on creative drives (i.e., mood, motivation, and emotion). Moreover, we discussed potential candidate genes for creativity.

Herewith we presented our perspective to advance our knowledge about creativity research through evaluating an overarching model of the interactions between creative cognition (i.e., cognitive flexibility, inhibitory control, WM updating, fluency, originality, and insights) and creative drive (i.e., emotion motivation, reward and other factors such as mood states, regulatory focus, social interaction), and the underlying neuromodulator mechanisms (Figure 1).

Lastly, we highlighted the possibility of implementing a neural network model as a predictive tool for the suggested integrated framework of creativity. For more insights on the computational model of creativity and emotion, see Perlovsky and Levine (2012) and Levine and Perlovsky (2011), respectively.

## Author Contributions

RK and BG outlined the structure of the review and wrote the manuscript. AK participated in the conceptualization of the manuscript and the final editing.

## Conflict of Interest Statement

The authors declare that the research was conducted in the absence of any commercial or financial relationships that could be construed as a potential conflict of interest.

## Abbreviations

5-HT: serotonin
ADs: antidepressants
ALE: activation likelihood estimation
BG: basal ganglia
BVSR: blind variation and selective retention
CAQ: Creative Achievement Questionnaire
CCI: composite creativity index
COMT: catechol-O-methyl-transferase
DA: dopamine
DAT: Dopamine Transporter
DMN: default mode network
DRD2: D2 Dopamine Receptor
DRD4: D4 Dopamine Receptor
DT: divergent thinking
EBR: spontaneous eye-blink rates
EFs: executive functions
FTD: frontotemporal dementia
mPFC: medial prefrontal cortex
mTG: middle temporal gyrus
NAc: nucleus accumbens
NE: noradrenaline
PCC: posterior cingulate cortex
PD: Parkinson’s disease
PFC: prefrontal cortex
RSFC: resting-state functional connectivity
STN: Substantia Nigra
TID: task-induced deactivation
TPH1: Tryptophan Hydroxylase
vlPFC: right ventrolateral prefrontal region
VTA: tegmental ventral area
WM: working memory.
